# Resistance Characteristics of SMA Actuator Based on the Variable Speed Phase Transformation Constitutive Model

**DOI:** 10.3390/ma13061479

**Published:** 2020-03-24

**Authors:** Yifan Lu, Rongru Zhang, Ye Xu, Lei Wang, Honghao Yue

**Affiliations:** 1 State Key Laboratory of Robotics and System, Harbin Institute of Technology, Harbin 150001, China; yf.lu@hit.edu.cn (Y.L.);; 2 School of Naval Architecture and Ocean Engineering, Harbin Institute of Technology, Weihai 264200, China

**Keywords:** shape memory alloy, phase transformation, constitutive model, resistance characteristics

## Abstract

The shape memory alloy (SMA)-based actuators have been increasingly used in different domains, such as automotive, aerospace, robotic and biomedical applications, for their unique properties. However, the precision control of such SMA-based actuators is still a problem. Most traditional control methods use the force/displacement signals of the actuator as feedback signals, which may increase the volume and weight of the entire system due to the additional force/displacement sensors. The resistance of the SMA, as an inherent property of the actuator, is a dependent variable which varies in accordance with its macroscopic strain or stress. It can be obtained by the voltage and the current imposed on the SMA with no additional measuring devices. Therefore, using the resistance of the SMA as feedback in the closed-loop control is quite promising for lightweight SMA-driven systems. This paper investigates the resistance characteristics of the SMA actuator in its actuation process. Three factors, i.e., the resistivity, the length, and the cross-sectional area, which affect the change of resistance were analyzed. The mechanical and electrical parameters of SMA were obtained using experiments. Numerical simulations were performed by using the resistance characteristic model. The simulation results reveal the change rules of the resistance corresponding to the strain of SMA and demonstrate the possibility of using the resistance for feedback control of SMA.

## 1. Introduction

The shape memory alloy (SMA) is a kind of alloy which can remember its original shape. When deformed by an external force, it can return to the pre-deformed shape by heating. SMAs have shape memory effect and superelasticity, as well as good physical and chemical properties and biocompatibility [1]. As the actuators based on SMA have the advantages of simple structure, small size, light quality, and low cost, the SMA is increasingly used in different fields [2]. For example, advanced SMA-based devices have been designed to improve the aerodynamic performance of vehicles [3], to actuate light grippers and reusable non-explosive lock release mechanisms [4,5], and to generate constant force components [6], etc.

However, how to accurately and effectively control the output force and displacement of the SMA is challenging. The resistance characteristics of SMA is important for the closed-loop control of SMA actuators. When displacement or force is used as the feedback signal in the control of the SMA, it is necessary to add a corresponding displacement sensor or force sensor. However, in practical engineering applications, the addition of external sensors will increase the volume of the structure and the whole process of detecting the state of the SMA cannot be realized effectively. Therefore, as an internal variable of SMA, the resistance can be considered as a feedback signal in the control. The resistance changes with the state of SMA in real time, and the measurement circuit can be quite small, which is more conducive to the practical application of SMA actuators [7]. In order to make the resistance of SMA an effective feedback variable, it is first necessary to establish the relationship between resistance and strain. The research on the resistance characteristics of SMA is usually based on the constitutive model of SMA and combined with the resistance law [8].

Furst et al. studied a single-shape memory alloy spring system by adding a second opposing shape memory alloy wire and focusing on the resistance to strain mapping that is crucial for self-sensing applications [9]. Zhu et al. applied this method to the self-repair of shape memory alloy composites to eliminate cracks and prevent overheating as soon as possible [10]. Nahm et al. studied the relationship between resistivity and tensile properties of Ni-Ti SMA wires for the process of superelasticity of SMA. The possibility of application of Ni-Ti SMA wire as a sensor was also studied. The sensor system can measure strain of 6%, and measurement error is 0.22%, and sensitivity of the rate of resistivity is 0.005% [11]. Yan et al. established a resistance–strain model of SMA for the process of superelasticity by using thermal dynamic and resistance theories. On the basis of this model, a piecewise linear model of the process of superelasticity was established through numerical analysis and experimental verification [12]. Cui et al. established a resistance characteristic model of Ni-Ti SMAs. The relationship between the change of the relative resistance and strain of SMA at different temperatures was analyzed, and the resistance characteristics of Ni-Ti SMA wires were tested to verify the validity of the model [13]. Also, Cui et al. analyzed the sensing principle based on the resistance of SMA. According to the relation between the resistivity of SMA wire and martensitic volume fraction, a mathematical model was established, which is applicable to different temperatures and initial conditions. The numerical simulation results based on the model are in good agreement with the experimental results, indicating that SMA wires with appropriate models can be used as sensing elements [14]. Xu et al. established the resistance characteristic model of Ni-Ti SMA fibers based on the mixed law of the martensite growth model and resistance. The relationship between resistivity and temperature of the fibers at zero stress was also studied [15]. Zhang et al. designed an embedded control circuit based on SMA internal resistance feedback. The heating control circuit of the SMA actuator and the resistance measurement and acquisition circuit of constant current source bridge method were built using an ATMEGA16 microcontroller. The experiment showed that the measurement method can achieve sufficient accuracy [16]. Lynch et al. proposed a stress–strain–resistance model. The model considers different loads, primary and secondary hysteresis effects, and is normalized according to the geometry of the actuator. The simulation results and a simple position control experiment were used to verify the performance of the model, and the correlation between the model and Liang’s enhancement model was proposed [17]. It can be seen that, in order to reflect the deformation of SMA via the change of resistance, many studies have been done on the resistance characteristic models of SMA [18,19]. The most common way to establish the resistance characteristic model is to use the constitutive model of SMA to reveal the relationship between resistance and strain [20]. Therefore, the accuracy of the constitutive model will directly determine the quality of the resistance model. For one-dimensional SMA actuators, the most commonly used constitutive model is the one-dimensional macro phenomenological constitutive model, because it introduces less internal variables and can easily predict the thermomechanical behaviors of SMA [21,22,23]. However, most of the existing resistance characteristic models are based on Liang’s model or Brinson’s model, which ignore the influence of the phase transformation rate distribution on the change of SMA’s resistivity [12,13,14,15,16,17]. Therefore, it is difficult to accurately predict the resistance characteristic of the SMA during the phase transformation process with a high degree of asymmetry.

In this paper, the resistance characteristic model of SMA is established from the perspective of the relative change of resistance, and the variable speed phase transformation constitutive model is used to acquire the parameters that affect the change of resistance. In this model, the effects of the resistivity, length and cross-sectional area of SMA on the relative change of resistance are fully considered. In order to simulate the relationship between resistance and strain, the characteristic parameters of the Ni-Ti SMA wire were identified using experiments, including the DSC experiment, the loading experiment and the thermal cycling experiment. The Ni and Ti contents of the SMA wire were 54.94 wt % and 45.06 wt %, respectively. Finally, the simulation results were analyzed and the possibility of using resistance for feedback control was proved.

## 2. Variable Speed Phase Transformation Constitutive Model

The phase composition of SMA changes with its stress and temperature. When the stress increases above the start critical stress (σ*_s_*), twinned martensite is transformed into detwinned martensite, and when the stress reaches the finish critical stress (σ*_f_*), the transformation is complete. However, when the stress decreases, the phase composition does not change. When the temperature increases to the austenite start temperature (*A_s_*), martensite (including twinned and detwinned martensite) is transformed into austenite, and when the temperature reaches the austenite finish temperature (*A_f_*), the transformation is complete. When the temperature decreases to the martensite start temperature (*M_s_*), the austenite is transformed into twinned martensite, and when the temperature reaches the martensite finish temperature (*M_f_*), the transformation is complete. In this study, a new variable speed phase transformation model is used to describe the phase composition change of SMA, including the phase transformation equation and the constitutive equation.

### 2.1. Phase Transformation Equation

In all the thermomechanical behaviors of SMA, the crystal phase transformation process of SMAs can be divided into three categories: martensite (M) to austenite (A), austenite (A) to martensite (M), and twinned martensite (TM) to detwinned martensite (DM). By referring to the derivation process of Liang and introducing the crystal variable speed function *K*, a new constitutive model was obtained in our previous research [23]. For a specific phase transformation process of the SMA, the crystal variable function *K* can be determined by the material parameters of SMA and a crystal variable speed coefficient *k*. The coefficient *k* is a constant determined by experiments, which reflects the rate distribution of the volume fraction of martensite/austenite with temperature or stress during the transformation process. Please note that all the variables and abbreviations mentioned in this article and their meanings are shown in Appendix A.

For the *M* → *A* transformation process, the volume fraction of austenite can be derived as

(1) $$\xi_{A}=1+(\xi_{A0}-1){\left\{\frac{1}{2}\left[\cos\frac{\pi (T-A_{s}-\sigma /C_{A})}{\left(A_{s}-A_{f}\right)}+1\right]\right\}}^{K_{1}}$$

where

(2) $$K_{1}=\left[1-\frac{\left(T-A_{s}-\sigma /C_{A})(k_{1}-1\right)}{(A_{f}-A_{s})k_{1}}\right]k_{1}$$

For the *A* → *M* transformation process, the volume fraction of austenite is derived as

(3) $$\xi_{A}=\xi_{A0}{\left\{\frac{1}{2}\left[\cos\frac{\pi (T-M_{s}-\sigma /C_{M})}{\left(M_{s}-M_{f}\right)}+1\right]\right\}}^{K_{2}}$$

where

(4) $$K_{2}=\left[1+\frac{\left(T-M_{s}-\sigma /C_{M})(k_{2}-1\right)}{(M_{s}-M_{f})k_{2}}\right]k_{2}$$

where *C_A_* and *C_M_* are coefficients related to the phase transformation critical stress and the phase transformation temperature of SMAs.

For the *TM* → *DM* transformation process, the volume fraction of detwinned martensite is derived as

(5) $$\xi_{DM}=1-\xi_{A}+\left(1-\xi_{A}\right)\left(\xi_{DM0}-1\right){\left\{\frac{1}{2}\left[\cos\frac{\pi (\sigma -\sigma_{s})}{\left(\sigma_{s}-\sigma_{f}\right)}+1\right]\right\}}^{K_{3}}$$

where

(6) $$K_{3}=\left[1-\frac{\left(\sigma -\sigma_{s})(k_{3}-1\right)}{(\sigma_{f}-\sigma_{s})k_{3}}\right]k_{3}$$

### 2.2. Constitutive Equation

The total strain of the SMA can be divided into three parts: the strain induced by elasticity, the strain induced by phase transformation and the strain induced by thermo [24]. According to the above strain classification, the constitutive equation can be derived as

(7) $$\varepsilon -\varepsilon_{0}=\frac{\sigma }{E(\xi_{A},\xi_{DM})}-\frac{\sigma_{0}}{E(\xi_{A}{}_{0},\xi_{DM0})}+\Psi \left(\xi_{DM}-\xi_{DM0}\right)+\alpha (\xi_{A})T-\alpha (\xi_{A0})T_{0}$$

where

(8) $$E(\xi_{A},\xi_{DM})=E_{TM}+(E_{A}-E_{TM})\xi_{A}+(E_{DM}-E_{TM})\xi_{DM}$$

(9) $$\alpha (\xi_{A})=\alpha_{M}+(\alpha_{A}-\alpha_{M})\xi_{A}$$

(10) $$\Psi =\varepsilon_{L}$$

**Figure 1:**
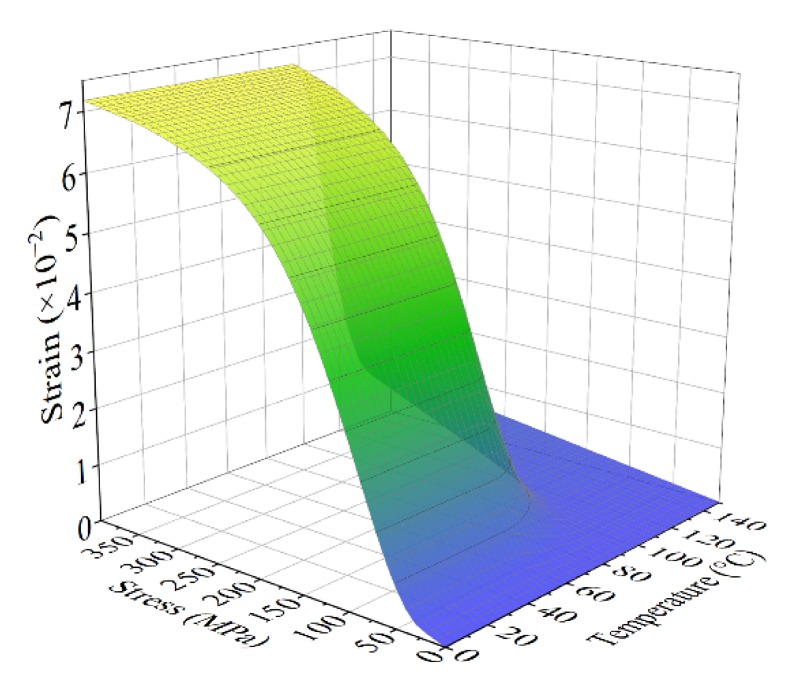
Relationship between temperature, stress, and strain of the shape memory alloy (SMA).

The volume fraction of each crystal phase in the transformation process was derived in Section 2.1. A numerical simulation of the constitutive model was performed with the initial state setting as *ε*_0_ = 0, *σ*_0_ = 0, *ξ_TM_*_0_ =1, *T*_0_ = 0 °C. The results are presented in Figure 1, which reveals the relationship between temperature, stress, and strain of the SMA. The parameters used in the simulation can be found in Table 1 and Table 2, which are identified by experiment.

With the above-mentioned constitutive model, the resistance characteristic model of SMA will be derived in the next section, which can predict the thermomechanical behaviors of SMA in accordance with its resistance.

## 3. Resistance Characteristic Model

In this part, a one-dimension SMA wire is considered. The resistance *R* of the SMA can be described as

(11) $$R=\frac{\rho l}{S}$$

where *ρ* is the resistivity, *l* is the length, and *S* is the cross-sectional area. By taking the natural logarithm of Equation (11), and then applying the total differential operator, the resistance equation can be derived as

(12) $$\frac{dR}{R}=\frac{d\rho }{\rho }+\frac{dl}{l}-\frac{dS}{S}$$

Thus, the three factors affecting the change of resistance of SMA are separated and can be expressed separately. Among them, the resistivity varies with temperature, which can be described as

(13) $$\rho =\rho_{0}\left(1+aT\right)$$

where *ρ*_0_ is the resistivity when temperature is 0 °C, and *a* is the temperature coefficient of resistance. Therefore, the resistivity equation of the decomposed form can be obtained as

(14) $$\frac{d\rho }{\rho }=\frac{d\rho_{0}}{\rho_{0}}+\frac{adT}{1+aT}$$

**Figure 2:**
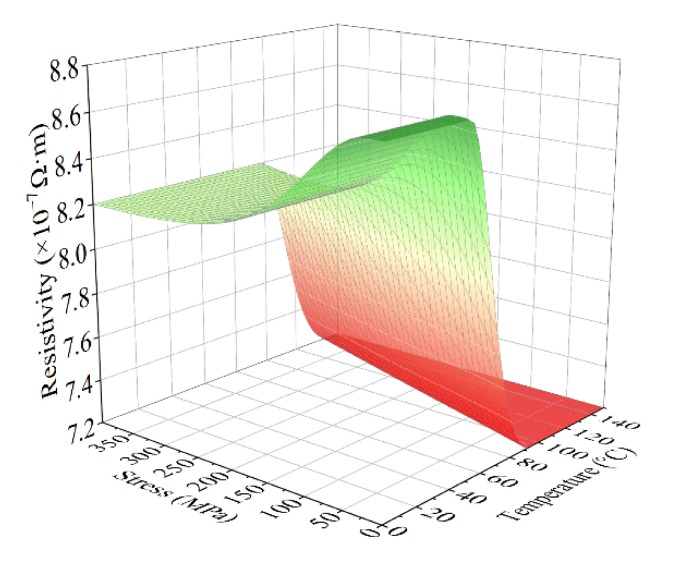
Relationship between temperature, stress and resistivity of the SMA.

Since *ρ*_0_ is affected by the volume fraction of twinned martensite, detwinned martensite and austenite, *ρ*_0_ can be described as

(15) $$\rho_{0}=\rho_{TM}+\left(\rho_{A}-\rho_{TM}\right)\xi_{A}+\left(\rho_{DM}-\rho_{TM}\right)\xi_{DM}$$

where *ρ_TM_* is the resistivity of twinned martensite, *ρ_DM_* is the resistivity of detwinned martensite, and *ρ_A_* is the resistivity of austenite. Since both *ξ_A_* and *ξ_DM_* are functions of temperature and stress, *ρ*_0_ is the function of temperature *T* and stress *σ*, too. Combining with the phase transformation equation of the constitutive model from Equations (1)–(6), the relation between *ρ*_0_, *T* and *σ* can be obtained. A numerical simulation of the resistivity of SMA was carried out. The relationship between temperature, stress, and resistivity of the SMA is shown in Figure 2. The parameters used in the simulation are collected in Table 1 and Table 2.

Substituting Equations (14) into Equations (12), the resistance equation is derived as

(16) $$\frac{dR}{R}=\frac{d\rho_{0}}{\rho_{0}}+\frac{adT}{1+aT}+\frac{dl}{l}-\frac{dS}{S}$$

where

(17) $$\frac{dS}{S}=\frac{2\pi rdr}{\pi r^{2}}=2\frac{dr}{r}=-2\nu \frac{dl}{l}$$

where *r* is the radius of the cross section, and *ν* is Poisson’s ratio. Convert Equation (16) into incremental form, the resistance characteristic model can be finally derived as

(18) $$\frac{\Delta R}{R}=\frac{\Delta \rho_{0}}{\rho_{0}}+\frac{a\Delta T}{1+aT}+\left(1+2\nu \right)\varepsilon$$

## 4. Parameter Identification

**Figure 3:**
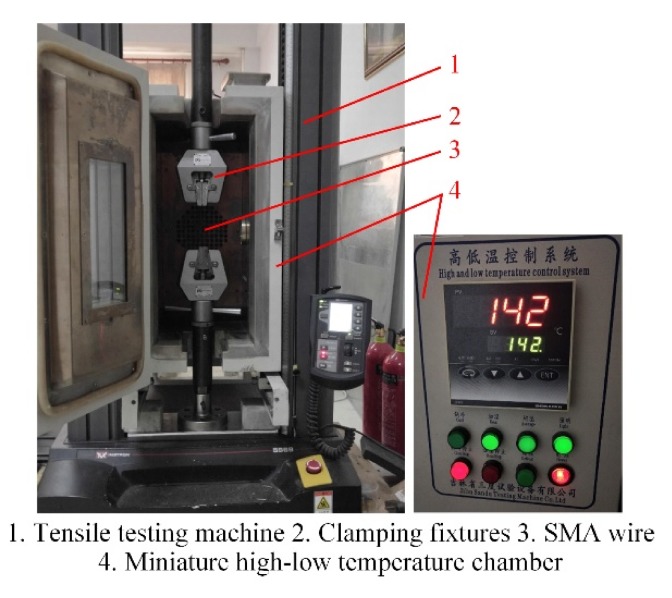
The loading experiments at 25 °C and 100 °C.

**Figure 4:**
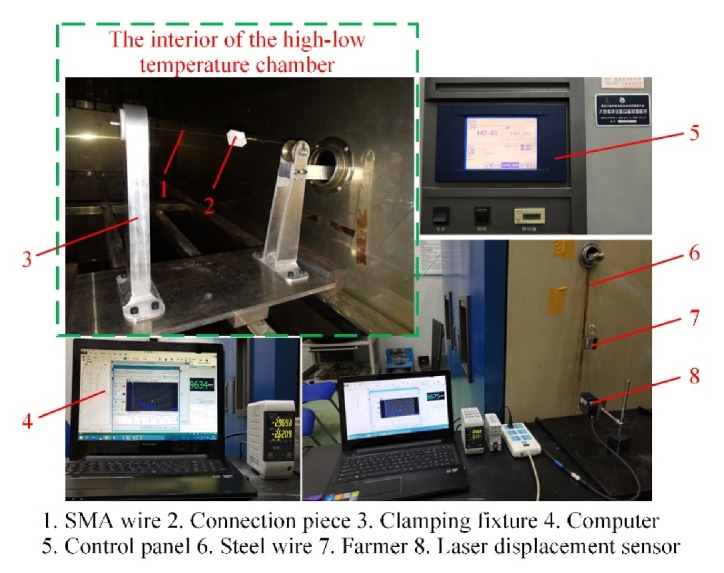
The thermal cycling experiments.

The parameters of the SMA wire are identified by experiments. The diameter of the SMA wire is 0.4 mm, with 54.94 wt % Ni and 45.06 wt % Ti. Firstly, the DSC experiment using a DSC synchronous thermal analyzer (LINSEIS STA PT1000) was performed, which gave the phase transformation temperatures, and the coefficients *k*_1_ and *k*_2_. Then, the loading experiments at 25 and 100 °C were performed with a tensile testing machine and a miniature high-low temperature chamber, as shown in Figure 3. The elastic moduli, critical stresses, maximum residual strain and the coefficient *k*_3_ were obtained. Finally, the thermal cycling experiment was conducted using a high-low temperature chamber (GDW/GDJS-100) and a laser displacement sensor (Keyence LK-G5000), as shown in Figure 4, based on which the coefficient *C_A_* as well as *C_M_* were obtained. The thermal and mechanical parameters of the SMA wire are collected in Table 1. The electrical parameters of the SMA were measured by an Agilent digital multimeter (344450A) and listed in Table 2.

## 5. Numerical Simulation

Numerical simulations of three cases were carried out: the loading experiments at 25 °C (100% martensite in the SMA), the loading experiments at 100 °C (100% austenite in the SMA), and the thermal cycling experiment. The parameters used in the numerical simulation are shown in Table 1 and Table 2. The initial condition of loading at 25 °C is ε_0_ = 0, σ_0_ = 0, *ρ*_0_ = *ρ_TM_*. Assuming that the temperature is constant during the experiment, the relative change in resistance in this case is

(19) $$\frac{\Delta R}{R_{r}}=\frac{\Delta \rho_{0}}{\rho_{TM}}+\left(1+2\nu \right)\varepsilon$$

where $\Delta \rho_{0}=\rho_{0}-\rho_{TM}$. Combined with the variable speed phase transformation constitutive model, the three-segment function of the loading process is

(20) $$\frac{\Delta R}{R_{r}}=\{\begin{array}{cc} \left(1+2\nu \right)\frac{\sigma }{E_{TM}}, & \sigma <\sigma_{s}\\ \frac{\rho_{DM}-\rho_{TM}}{\rho_{TM}}\xi_{DM}+\left(1+2\nu \right)\left(\frac{\sigma }{E\left(\xi_{A},\xi_{DM}\right)}+\Psi \xi_{DM}\right), & \sigma_{s}\leq \sigma <\sigma_{f}\\ \frac{\rho_{DM}-\rho_{TM}}{\rho_{TM}}+\left(1+2\nu \right)\left(\frac{\sigma }{E_{DM}}+\varepsilon_{L}\right), & \sigma \geq \sigma_{f} \end{array}$$

**Figure 5:**
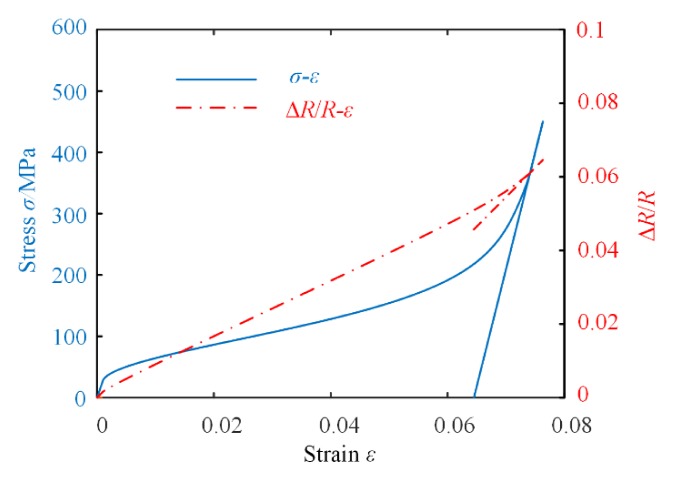
The relationship between *σ*-*ε* and *σ*-Δ*R*/*R* at 25 °C.

The function of the unloading process is

(21) $$\frac{\Delta R}{R_{r}}=\frac{\rho_{DM}-\rho_{TM}}{\rho_{TM}}+\left(1+2\nu \right)\left(\frac{\sigma }{E_{DM}}+\varepsilon_{L}\right)$$

where ξ*_DM_*, *E*(ξ*_A_*, ξ*_DM_*), and Ψ are obtained from Section 2. The relative change of resistance Δ*R*/*R_r_* can be simulated by MATLAB. Combined with the simulation results of strain *ε*, the relations between Δ*R*/*R_r_* and *ε* are plotted in Figure 5.

The initial condition of loading at 100 °C is *ε*_0_ = *α_A_T*_0_ − *α_M_T_r_*, *σ*_0_ = 0, *ρ*_0_ = *ρ_A_*. Assuming that the temperature is constant during the experiment, the relative change in resistance in this case is

(22) $$\frac{\Delta R}{R_{r}}=\frac{\Delta \rho_{0}}{\rho_{TM}}+\left(1+2\nu \right)\left(\varepsilon -\varepsilon_{0}\right)$$

where $\Delta \rho_{0}=\rho_{0}-\rho_{A}=\rho_{TM}+\left(\rho_{A}-\rho_{TM}\right)\xi_{A}+\left(\rho_{DM}-\rho_{TM}\right)\xi_{DM}-\rho_{A}$. Combined with the variable speed phase transformation constitutive model, the three-segment function of the loading process is

(23) $$\frac{\Delta R}{R_{r}}=\{\begin{array}{cc} \left(1+2\nu \right)\frac{\sigma }{E_{A}}, & \sigma <C_{M}\left(T-M_{s0}\right)\\ \frac{\Delta \rho_{0}}{\rho_{TM}}+\left(1+2\nu \right)\Delta \varepsilon , & \;C_{M}\left(T-M_{s0}\right)\leq \sigma <C_{M}\left(T-M_{f0}\right)\;\\ \frac{\rho_{DM}-\rho_{A}}{\rho_{TM}}+\left(1+2\nu \right)\left(\frac{\sigma }{E_{DM}}+\Psi +\alpha_{M}T_{0}-\alpha_{A}T_{0}\right), & \sigma \geq C_{M}\left(T-M_{f0}\right) \end{array}$$

**Figure 6:**
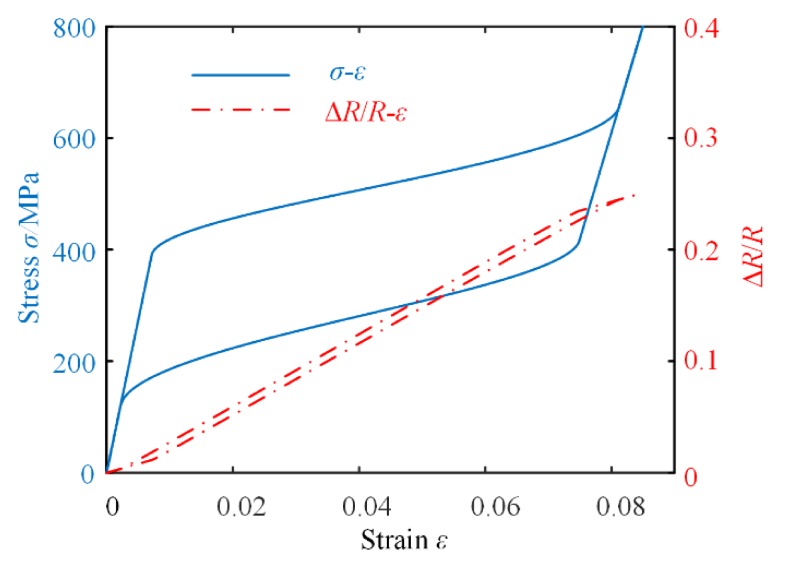
The relationship between *σ*-*ε* and *σ*-Δ*R*/*R* at 100 °C.

The three-segment function of the unloading process is

(24) $$\frac{\Delta R}{R_{r}}=\{\begin{array}{cc} \frac{\rho_{DM}-\rho_{A}}{\rho_{TM}}+\left(1+2\nu \right)\left(\frac{\sigma }{E_{DM}}+\Psi +\alpha_{M}T_{0}-\alpha_{A}T_{0}\right), & \sigma \geq C_{A}\left(T-A_{s0}\right)\\ \frac{\Delta \rho_{0}}{\rho_{TM}}+\left(1+2\nu \right)\Delta \varepsilon , & C_{A}\left(T-A_{f0}\right)\leq \sigma <C_{A}\left(T-A_{S0}\right)\;\\ \left(1+2\nu \right)\frac{\sigma }{E_{A}}, & \sigma <C_{A}\left(T-A_{f0}\right) \end{array}$$

where

(25) $$\Delta \varepsilon =\frac{\sigma }{E(\xi_{A},\xi_{DM})}+\Psi \xi_{DM}+\alpha (\xi_{A})T_{0}-\alpha_{A}T_{0}$$

where ξ*_A_*, ξ*_DM_*, *E*(ξ*_A_*, ξ*_DM_*), α(ξ*_A_*), and Ψ are obtained from Section 2. The relative change in resistance Δ*R*/*R_r_* can be simulated by MATLAB. Combined with the simulation results of strain ε, the relations between Δ*R*/*R_r_* and ε are presented in Figure 6.

The initial condition of the thermal cycling experiment is *σ*_0_ = 117 MPa, *T*_0_ =25 °C (*T*_0_ < *M_f_*_0_), *ξ_A_*_0_ = 0. Combining constitutive Equation (7) with phase transformation Equation (5), *ε*_0_ is

(26) $$\varepsilon_{0}=\frac{\sigma_{0}}{E\left(\xi_{A0},\xi_{DM0}\right)}+\Psi \xi_{DM0}$$

(27) $$\xi_{DM0}=1-{\left\{\frac{1}{2}\left[\cos\frac{\pi (\sigma_{0}-\sigma_{s})}{\left(\sigma_{s}-\sigma_{f}\right)}+1\right]\right\}}^{K_{3}}$$

The stress changed little during the whole experiment, so it can be assumed that the stress was constant. The relative change of resistance is

(28) $$\frac{\Delta R}{R_{r}}=\frac{\Delta \rho_{0}}{\rho_{TM}}+\frac{a\Delta T}{1+aT_{0}}+\left(1+2\nu \right)\left(\varepsilon -\varepsilon_{0}\right)$$

where $\Delta \rho_{0}=\rho_{0}-\rho_{00}=\left(\rho_{A}-\rho_{TM}\right)\left(\xi_{A}-\xi_{A0}\right)+\left(\rho_{DM}-\rho_{TM}\right)\left(\xi_{DM}-\xi_{DM0}\right)$.

Combined with the variable speed phase transformation constitutive model, the three-segment function of the heating process is

(29) $$\frac{\Delta R}{R_{r}}=\left\{ \begin{array}{cc} \frac{a\left(T-T_{0}\right)}{1+aT_{0}}+\left(1+2\nu \right)\alpha_{M}\left(T-T_{0}\right), & T_{0}<T<\frac{\sigma_{0}}{C_{A}}+A_{s}\\ \frac{\Delta \rho_{0}}{\rho_{TM}}+\frac{a\left(T-T_{0}\right)}{1+aT_{0}}+\left(1+2\nu \right)\Delta \varepsilon_{1}, & \frac{\sigma_{0}}{C_{A}}+A_{s}\leq T<\frac{\sigma_{0}}{C_{A}}+A_{f}\\ \frac{\Delta \rho_{0}}{\rho_{TM}}+\frac{a\left(T-T_{0}\right)}{1+aT_{0}}+\left(1+2\nu \right)\Delta \varepsilon_{2}, & T\geq \frac{\sigma_{0}}{C_{A}}+A_{f} \end{array}\right.$$

**Figure 7:**
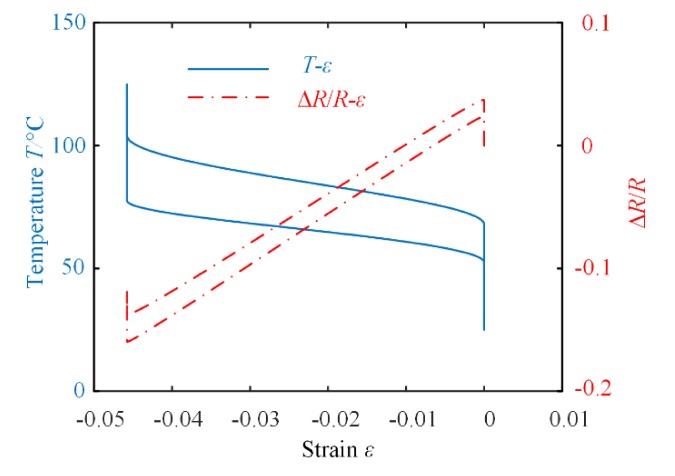
The relationship between *T*-*ε* and *σ*-Δ*R*/*R* at 117 MPa.

The three-segment function of the cooling process is

(30) $$\frac{\Delta R}{R_{r}}=\left\{ \begin{array}{cc} \frac{\Delta \rho_{0}}{\rho_{TM}}+\frac{a\left(T-T_{0}\right)}{1+aT_{0}}+\left(1+2\nu \right)\Delta \varepsilon_{2}, & T\geq \frac{\sigma_{0}}{C_{M}}+M_{s}\\ \frac{\Delta \rho_{0}}{\rho_{TM}}+\frac{a\left(T-T_{0}\right)}{1+aT_{0}}+\left(1+2\nu \right)\Delta \varepsilon_{1}, & \frac{\sigma_{0}}{C_{M}}+M_{f}\leq T<\frac{\sigma_{0}}{C_{M}}+M_{s}\\ \frac{a\left(T-T_{0}\right)}{1+aT_{0}}+\left(1+2\nu \right)\alpha_{M}\left(T-T_{0}\right), & T_{0}<T<\frac{\sigma_{0}}{C_{,M}}+M_{f} \end{array}\right.$$

where

(31) $$\Delta \varepsilon_{1}=\frac{\sigma_{0}}{E(\xi_{A},\xi_{DM})}-\frac{\sigma_{0}}{E(\xi_{A}{}_{0},\xi_{DM0})}+\Psi \left(\xi_{DM}-\xi_{DM0}\right)+\alpha (\xi_{A})T-\alpha_{M}T_{0}$$

(32) $$\Delta \varepsilon_{2}=\frac{\sigma_{0}}{E_{A}}-\frac{\sigma_{0}}{E(\xi_{A}{}_{0},\xi_{DM0})}-\Psi \xi_{DM0}+\alpha_{A}T-\alpha_{M}T_{0}$$

where ξ*_A_*, ξ*_DM_*, *E*(ξ*_A_*, ξ*_DM_*), *E*(ξ*_A_*_0_, *ξ_DM_*_0_), α(ξ*_A_*), and Ψ are obtained from Section 2. The relative change in resistance Δ*R*/*R_r_* can be simulated by MATLAB. Combined with the simulation results of strain *ε*, the relations between Δ*R*/*R_r_* and *ε* are depicted in Figure 7.

It can be observed from Figure 5 to Figure 7 that for different cases, the relative change in resistance of the SMA varies corresponding to the strain. In fact, for most applications of SMA-based actuators, we just need to pay close attention to a particular phase transition process of the SMA. Thus, it is possible to monitor the resistance instead of the strain of the SMA for precision control of the SMA-based actuator. The proposed SMA resistance characteristic model can therefore be used to accurately calculate the output displacement or force of the actuator.

## 6. Conclusions

A new resistance characteristic model of SMA was proposed in this work based on the variable speed phase transformation constitutive model. The influences of different parameters, such as the resistivity, the length, the cross-sectional area of the SMA, and the temperature on the resistance, were fully considered. The coupling relationship of stress–strain–temperature–resistance of SMA was revealed. Compared with the existing models, the proposed model further considers the influence of the phase transformation rate on the change of SMA’s resistivity, which can greatly enhance the accuracy of the model, especially for those with distinct asymmetry phase transformation process. Therefore, the proposed model can describe the one-to-one correspondence between resistance and strain of SMA in a particular phase transformation process more accurately and comprehensively; namely, the resistance can be used as an internal variable to reflect the strain of SMA. The model serves as an important theoretical basis for precision control of SMA-based actuator with resistance signal feedback, which will further promote the study on the control of SMA-based systems, and is conducive to the lightweight and miniaturization of SMA-based structures.

## Figures and Tables

**Table 1:** Thermal and mechanical parameters of the SMA.

***M_f_* (°C)**	***M_s_* (°C)**	***A_s_* (°C)**	***A_f_* (°C)**	***σ_s_* (MPa)**	***σ_f_* (MPa)**
35.8	62.8	47.6	81.9	26.5	326.8
***E_A_* (GPa)**	***E_TM_* (GPa)**	***E_DM_* (GPa)**	***C_M_* (MPa/°C)**	***C_A_* (MPa/°C)**	***ε_L_***
54.64	17.96	31.52	10.4	8.0	0.0645
***α_M_* (°C^−1^)**	***α_A_* (°C^−1^)**	***l*_0_ (mm)**	***k*_1_**	***k*_2_**	***k*_3_**
2.73 × 10^−7^	9.16 × 10^−7^	100	1.15	1.28	7.88

**Table 2:** Electrical parameters of the SMA.

*ν*	*a* (°C^−1^)	*ρ_TM_* (Ω∙m)	*ρ_DM_* (Ω∙m)	*ρ_A_* (Ω∙m)
0.3	8.75 × 10^−4^	0.87 × 10^−6^	0.82 × 10^−6^	0.72 × 10^−6^

## Appendix A

**Table A1:** Nomenclature.

Symbol	Meaning	Symbol	Meaning
*T*	Temperature	*k*	Crystal variable speed coefficient
*M_s_*	Martensite start temperature	*K*	Crystal variable speed function
*M_f_*	Martensite finish temperature	*A*	Austenite
*A_s_*	Austenite start temperature	*DM*	Detwinned martensite
*A_f_*	Austenite finish temperature	*TM*	Twinned martensite
*M_p_*	Martensite peak temperature	*Ψ*	Transformation modulus
*A_p_*	Austenite peak temperature	*E_A_*	Elastic modulus of austenite
*σ*	Stress	*E_TM_*	Elastic modulus of *TM*
*σ_s_*	Starting stress	*E_DM_*	Elastic modulus of *DM*
*σ_f_*	Finishing stress	*ξ_A_*	Volume fraction of austenite
*σ_p_*	Peaking stress	*ξ_DM_*	Volume fraction of *DM*
*ε*	Strain	*ε_L_*	Maximum residual strain
*α_M_*	Thermal expansion coefficient of martensite	*α_A_*	Thermal expansion coefficient of austenite
*ν*	Poisson’s ratio	*a*	Temperature coefficient of *R*
*S*	Cross-sectional area.	*l*	Length
*ρ*	Resistivity	*ρ_TM_*	Resistivity of *TM*
*Ρ_DM_*	Resistivity *DM*	*ρ_A_*	Resistivity *DM*
